# Neuroprotective and Cognitive Enhancement Potentials of Baicalin: A Review

**DOI:** 10.3390/brainsci8060104

**Published:** 2018-06-11

**Authors:** Kandhasamy Sowndhararajan, Ponnuvel Deepa, Minju Kim, Se Jin Park, Songmun Kim

**Affiliations:** School of Natural Resources and Environmental Science, Kangwon National University, Chuncheon 24341, Gangwon-do, Korea; taanishadeepa@gmail.com (P.D.); camin1121@gmail.com (M.K.); sejinpark@kangwon.ac.kr (S.J.P.)

**Keywords:** baicalin, cognitive, neurodegenerative disease, neuroprotective, *Scutellaria baicalensis*

## Abstract

Neurodegenerative diseases are a heterogeneous group of disorders that are characterized by the gradual loss of neurons. The development of effective neuroprotective agents to prevent and control neurodegenerative diseases is specifically important. Recently, there has been an increasing interest in selecting flavonoid compounds as potential neuroprotective agents, owing to their high effectiveness with low side effects. Baicalin is one of the important flavonoid compounds, which is mainly isolated from the root of *Scutellaria baicalensis* Georgi (an important Chinese medicinal herb). In recent years, a number of studies have shown that baicalin has a potent neuroprotective effect in various in vitro and in vivo models of neuronal injury. In particular, baicalin effectively prevents neurodegenerative diseases through various pharmacological mechanisms, including antioxidative stress, anti-excitotoxicity, anti-apoptotic, anti-inflammatory, stimulating neurogenesis, promoting the expression of neuronal protective factors, etc. This review mainly focuses on the neuroprotective and cognitive enhancement effects of baicalin. The aim of the present review is to compile all information in relation to the neuroprotective and cognitive enhancement effects of baicalin and its molecular mechanisms of action in various in vitro and in vivo experimental models.

## 1. Introduction

*Scutellaria baicalensis* Georgi (Lamiaceae or Labiatae) is an important Chinese traditional medicinal plant, and commonly known as Huang-Qin or Chinese skullcap (*Radix scutellariae*). Traditionally, the root part of this plant has been used for the treatment of various ailments, including diarrhea, hepatitis, atherosclerosis, dysentery, diabetes, hypertension, complication of menstruation, eye diseases, vomiting, hemorrhaging, insomnia, common cold, inflammation, and respiratory infections [1]. Further, several scientific studies have reported that extracts and isolated compounds from *S. baicalensis* have various pharmacological properties, such as antitumor, hepatoprotective, antimicrobial, anti-inflammatory, neuroprotective, anti-hyperlipidemic, antidiabetic, antimutagenesis, anticonvulsant, and antioxidant. So far, more than 50 flavonoid components have been isolated and identified from *S. baicalensis*. Among them, baicalin and its aglycone baicalein are the major bioactive compounds [2,3].

**Figure 1:**
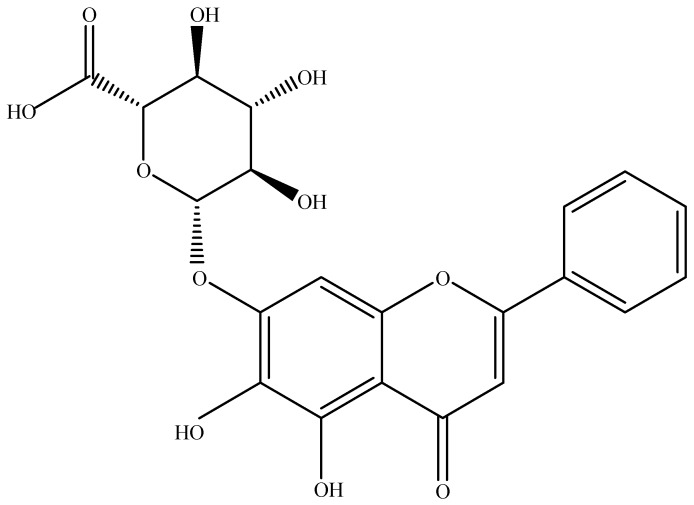
The chemical structure of baicalin.

Baicalin (C_21_H_18_O_11_; 5,6,7-trihydroxyflavone 7-*O*-beta-d-glucuronide or baicalein 7-*O*-β-d-glucuronic acid or 7-d-glucuronic acid-5,6-dihydroxyflavone) (Figure 1) is one of the most important flavonoid components in the roots of *Scutellaria baicalensis* Georgi. The concentration of baicalin in the roots of *S. baicalensis* ranged from 8.1% to 15.6% [4,5,6,7]. The root of this plant has been traditionally used for the treatment of numerous diseases in East Asia. Synonyms of *S. baicalensis* are *S. grandiflora* Adams, *S. lanceolaria* Miq., and *S. macrantha*. Baicalin is also the major constituent in other *Scutellaria* species, namely *S. lateriflora*, *S. galericulata*, and *S. rivularia*, as well as in *Oroxylum indicum* (Bignoniaceae). Different species of *Scutellaria* are mainly distributed in Asian countries, such as China, Russia, Mongolia, Japan, and Korea [8].

Baicalin has been extensively used in pharmaceutical and food industries due to its outstanding bioactivities. A number of in vitro and in vivo studies have demonstrated that baicalin possesses various pharmacological properties, including anti-inflammatory, anticancer, antidiabetic, antithrombotic, cardioprotective, hepatoprotective, and neuroprotective properties [8,9]. In particular, baicalin exhibits a variety of beneficial effects in the central nervous system (CNS) by promoting neural differentiation and inhibiting neuronal apoptosis [10]. Further, baicalin also shows antidepressant- and anxiolytic-like properties and improving cognitive performances [6]. Previous studies have demonstrated that baicalin showed protective effect against amyloid-β (Aβ), hydrogen peroxide (H_2_O_2_), oxygen/glucose deprivation (OGD), middle cerebral artery occlusion (MCAO), ketamine, and thrombin-induced neurotoxicity in cell lines and animal models [11,12,13,14,15,16].

The brain and nervous system-related diseases affect over two billion people worldwide and many of the most harmful brain diseases are neurodegenerative. Chronic neurodegenerative diseases, including Alzheimer’s (AD), Parkinson’s (PD), and Huntington’s diseases represent a large unmet medical need in the world. These diseases refer to those conditions in which neuronal cells undergo progressive degeneration and eventual death associated with aging [17]. Accumulating evidence suggests that excitotoxicity, oxidative stress, mitochondrial dysfunction, inflammatory response, protein misfolding, and neuronal apoptosis have been associated with neurodegeneration [9,18]. The most common symptoms of neurodegenerative diseases are anxiety, apathy, depression, motor dysfunction, and memory impairment [19]. Numerous studies have shown that baicalin efficiently prevents various neurodegenerative diseases, such as AD, PD, and cerebral ischemia, by suppressing oxidative stress, inhibiting excitotoxicity, and promoting neurogenesis, reducing apoptosis, and inhibiting the production of inflammatory cytokines [9,20,21].

Previously, some authors clearly reviewed therapeutic potentials of baicalin against cancer, cerebral ischemia, tumor, cardiovascular, ocular, and inflammatory disorders [5,8,9,22,23,24,25]. However, the low aqueous solubility and poor oral bioavailability of baicalin are the major limitations in the clinical application. Baicalin showed limited permeability, probably owing to its comparatively high hydrophilicity and larger molecular weight. In addition, baicalin was found to be moderately absorbed in the stomach and poorly in the small intestine and colon [26]. Moreover, previous studies clearly demonstrated that baicalin can be detected in the blood of animals immediately after oral or intravenous administration of baicalein [27,28]. In regard to drug–drug interaction, baicalin may influence the metabolism of several CYP2E1 isozyme dependent drugs [29]. Previous study reported that following treatment with aminoglycoside, the absolute bioavailability of baicalin was decreased by nearly 40–45% when compared with untreated rats. Some studies exhibited that baicalin could interact with transporter-related drugs (rosuvastatin, and 7-ethyl-10-hydroxycamptothecin) and other drugs such as cyclosporine A, quinidine, and SKF-525A [9].

**Figure 2:**
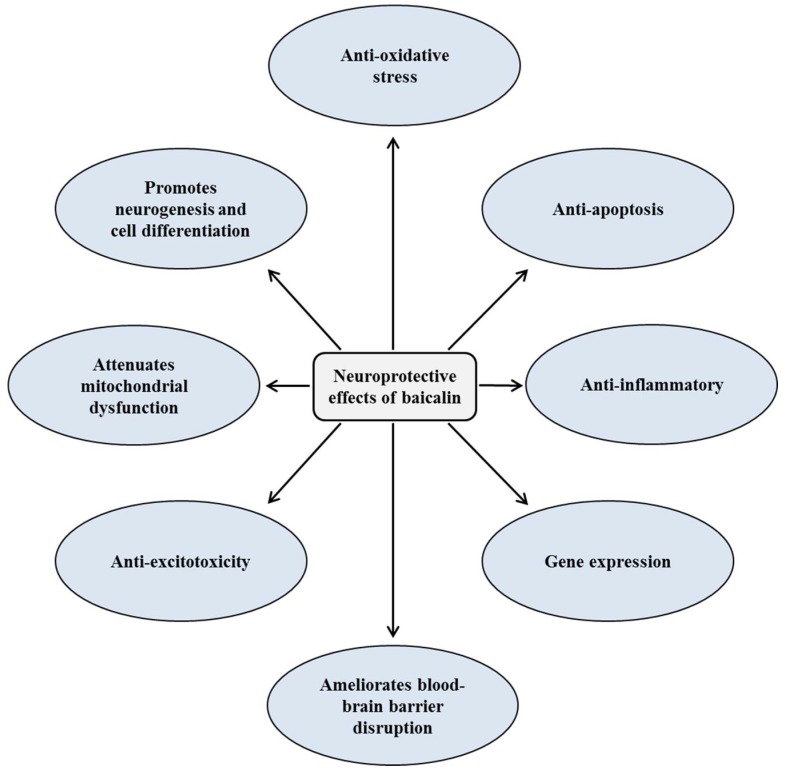
Mechanisms of neuroprotective and cognitive enhancement effects of baicalin.

There has been no specific review of published articles in relation to neuroprotective and cognitive enhancement effects of baicalin. Herein, we reviewed the protective effects of baicalin on various neurodegenerative disorders. Current knowledge about neuroprotective and cognitive enhancement properties of baicalin and its molecular mechanisms are presented in Table 1 and Table 2. Figure 2 illustrates the role of different signaling pathways in protecting neuronal damage.

## 2. Neuroprotective Effects of Baicalin

### 2.1. Alzheimer’s Disease Model

#### 2.1.1. In Vitro

The aggregation of Aβ peptides is an important factor in the etiopathogenesis of AD. Increasing evidence demonstrated that metal ions play a major role in the aggregation of Aβ. Hence, inhibition of Aβ aggregation appears as a potential strategy to treat AD. A study reported that baicalin can interact with copper directly and suppress Aβ_1-42_ aggregation. Further, baicalin showed a protective effect against Aβ_1-42_-induced oxidative injuries in SH-SY5Y cells by inhibiting the production of H_2_O_2_ [35]. Xiong et al. [19] determined whether baicalin regulates microglial activation or inhibits inflammatory cytokine production in Aβ_42_-induced BV2 cells. The results revealed that baicalin inhibited Aβ_42_-induced BV2 microglial cell proliferation, reduced cluster of differentiation molecule 11b (CD11b) expression, decreased the chemotactic ability of BV2 cells. In addition, baicalin effectively inhibited the production of interleukin-6 (IL-6), tumor necrosis factor-α (TNF-α), and nitric oxide (NO). Furthermore, baicalin pretreatment could significantly downregulate Aβ-induced phosphorylation of Janus kinase 2 (JAK2), and signal transducer and activator of transcription 3 (STAT3).

#### 2.1.2. In Vivo

A study was conducted to investigate therapeutic effects of a mixture of Chinese herb active components (baicalin, jasminoidin, and cholic acid) against ibotenic acid-induced rats. Pretreatment with the chemical mixture markedly attenuated abnormalities in cognition, brain functional images, and histological morphology in ibotenic acid-induced rats. The chemical mixture significantly influenced on the expression levels of 19 genes in the forebrain. In these, about 60% of genes were associated with neuroprotection and neurogenesis, and the remaining genes were linked with antioxidation, degradation of proteins, cholesterol metabolism, stress response, and apoptosis. In addition, the treatment increased the expression of these genes with the exception of the apoptosis-related gene [74]. Chen et al. [78] examined the neuroprotective effects of baicalin on pathological changes and behavioral impairments in Aβ_1-42_-induced AD mouse model. Baicalin could ameliorate memory impairment in the Morris water maze and probe tests. Further, baicalin treatment attenuated glial cell activation and decreased the expression of TNF-α and IL-6 in Aβ_1-42_-induced AD mice.

In Aβ-induced AD transgenic mice, baicalin significantly regulated the JAK2/STAT3 signaling pathway [14]. In an Aβ toxicity rat model of AD, baicalin treatment improved learning and memory deficits in Morris water maze test, and attenuated the hippocampus injury. Baicalin treatment significantly increased the level of antioxidant enzymes and their gene expressions, such as superoxide dismutase (SOD), catalase, and glutathione peroxidase (GSH-Px). In addition, baicalin increased mitochondrial membrane potential and reduced Bax/B cell lymphoma 2 family (Bcl-2) ratio, cytochrome c release, and caspase-9/-3 activation in Aβ-induced rats. Further, the antioxidative effect of baicalin may associate with the activation of nuclear factor erythroid 2-related factor 2 (Nrf2) signaling pathway [79]. Bitto et al. [85] investigated the neuroprotective effect of flavocoxid (a mixture of purified baicalin and catechin) in triple-transgenic (3xTg-AD) mice. Flavocoxid is a dual inhibitor of cyclooxygenases-1/2 (COX-1/2) and 5-lipoxygenase (5-LOX). Flavocoxid treatment improved learning and memory function, and decreased the production of eicosanoid. In addition, flavocoxid downregulated the activation of nucleotide-binding domain and leucine-rich repeat protein-3 (NLRP3) inflammasome and IL-1β production. Furthermore, flavocoxid reduced the phosphorylation level of the amyloid precursor protein (APP-p-Thr668), p-tau (p-Thr181) and extracellular signal-regulated kinases 1 and 2 (p-ERK1/2). A recent study demonstrated that baicalin increased the percentages of astrocytes and neuronal in Aβ_1-40_-induced rats. Moreover, baicalin treatment enhanced expressions of Nestin and nucleotide sugar epimerase [91].

### 2.2. Parkinson’s Disease Model

#### In Vivo

1-Methyl-4-phenyl-1,2,3,6-tetrahydropyridine (MPTP) is a potent and selective nigrostriatal dopaminergic neurotoxin that produces many of the neuropathological characteristics of idiopathic PD. In MPTP-induced mice, baicalin treatment effectively prevented the loss of tyrosine hydroxylase (TH)-positive neurons in substantia nigra, and increased the dopamine content of striatum. In addition, baicalin significantly increased glutathione content (GSH) in the brain [57]. Previous studies reported that iron accumulates in the substantia nigra of patients with PD. Xiong et al. [69] found that baicalin treatment markedly inhibited iron accumulation in different brain regions, and showed a protective effect on dopaminergic neurons in rotenone-induced rats.

### 2.3. Ischemia

#### 2.3.1. In Vitro

Ischemic stroke is the third most common cause of death worldwide in adults. According to the World Health Organization, 5–6 million individuals die from stroke each year [9]. OGD is a widely used in vitro model to study ischemic stroke. Liu et al. [30] investigated the effect of baicalin on ischemic-like or excitotoxic injury, and protein kinase C alpha (PKC(α)) activation in rat hippocampal slices. In OGD- and *N*-methyl-d-aspartate (NMDA)-induced rat hippocampal slices, baicalin ameliorated viability reduction and inhibited the increased membrane portion of PKC(α). It was reported that the activation of 5-LOX is involved in ischemic neuronal damage. Baicalin showed the protective effect against OGD-induced ischemic-like injury in rat cortical neurons by partly inhibiting NMDA receptor-mediated 5-LOX activation [31]. Jung et al. [32] found that baicalin attenuated the negative insult of light, hydrogen peroxide, and serum withdrawal to RGC-5 cells.

Nucleotide-binding oligomerization domain protein 2 (NOD2) receptor plays a key role in innate immunity, and is genetically associated with various inflammatory reactions. In this context, the effect of baicalin on NOD2/TNFα in OGD-induced BV2 cells, PC12 cells, and primary neuron cells was investigated. The results indicated that baicalin treatment could effectively suppress the expression of NOD2 and TNFα in mRNA as well as protein levels [34]. In OGD-induced microglial cells, baicalin markedly inhibited the release of TNF-α, IL-1β, IL-6, and IL-8, and reduced Toll-like receptor 4 (TLR4) mRNA expression and tumor necrosis factor receptor-associated factor 6 (TRAF6) levels. Additionally, baicalin downregulated nuclear factor of kappa light polypeptide gene enhancer in B-cells inhibitor-alpha (IκB-α), c-jun, ERK1/2, c-Jun N-terminal kinase (JNK), and p38 phosphorylation, and inhibited the transfer of myeloid differentiation primary response 88 (MyD88) from cytoplasm to membrane in OGD-induced microglial cells [36].

In another study, baicalin effectively protected neurons against H_2_O_2_-induced injury and improved the SOD activity [38]. Baicalin also showed the neuroprotective effect against extraneous and endogenous peroxynitrite-induced toxicity in SH-SY5Y cells [40]. Zhang et al. [41] examined the protective effect of baicalin on OGD-induced brain microvascular endothelial cells through anti-inflammation. The results showed that baicalin increased cell viability, and decreased lactate dehydrogenase (LDH) leakage rate, TNF-α, IL-1β, and IL-6 levels in the culture media. In addition, baicalin markedly downregulated the phosphorylation of proteins in mitogen-activated protein kinase (MAPK) signaling pathways, such as p-MRK1/2, p-ERK, and p-p38. Further, baicalin significantly downregulated the phosphorylation of proteins in nuclear factor kappa B (NF-кB) signaling pathway, such as phosphorylated-IκB kinase α and β (p-IKKα and p-IKKβ), and p-IκBα. Moreover, baicalin remarkably inhibited nuclear transcriptional activity triggered by NF-κB p65 and p-IκBα in brain microvascular endothelial cells. On the basis of these findings, authors suggested that baicalin exhibited its neuroprotective effect by downregulating the MAPK and NF-κB signaling pathway.

In H_2_O_2_-induced rat primary cortical neurons, baicalin effectively inhibited neuronal apoptosis by enhancing transcription and expression of myeloid cell leukemia-1 (MCL-1) and BCL-2. Additionally, baicalin remarkably increased myocardin-related transcription factor-A (MRTF-A) level. However, the anti-apoptosis effect of baicalin was significantly abrogated by the transfection of small interfering RNA of MRTF-A (MRTF-A siRNA) in primary cortical neuron cultures. Baicalin also increased the transactivity of MCL-1 and BCL-2 promoter by activating the key CarG box (CC [A/T] 6GG) element. Moreover, baicalin-induced MRTF-A expression and transactivity and expression of MCL-1 and BCL-2 were reduced by phosphatidylinositol-3 kinase (PI3K) inhibitor, LY294002, and ERK1/2 inhibitor, PD98059 [21]. Another study indicated that baicalin showed neuroprotective effects against hypoxia and OGD/reoxygenation (RO)-induced injury in SH-SY5Y cells. Baicalin significantly attenuated OGD/RO-induced apoptotic cell death in SH-SY5Y cells, and decreased caspase-3 expression. In addition, baicalin markedly downregulated NF-κB and *N*-methyl-d-aspartic acid receptor-1 (NMDAR1) in OGD/RO-induced cells [45].

A recent study reported that baicalin treatment suppressed dynamin-related protein 1 (Drp-1) expression, reduced mitochondrial fission, elevated mitofusin-2 (MFN2) generation, upregulated Drp-1 Ser637 phosphorylation, and increased mitochondrial membrane potential in OGD/reperfusion-induced PC12 cells via the inhibition of reactive oxygen species (ROS) production. In addition, baicalin attenuated cell apoptosis and increased mitophagy [13]. Luo et al. [48] evaluated the protective effect of baicalin against OGD-induced ischemic injury in endothelial cells. The authors found that baicalin effectively inhibited cell death, decreased cell membrane damage, and maintained the integrity of the nucleus. Baicalin treatment also decreased necroptosis ratio and regulated the expression of RIP-1 and RIP-3 in bEnd.3 cells. Furthermore, baicalin inhibited the production of ROS and malondialdehyde (MDA), and increased SOD activity in OGD-induced bEnd.3 cells.

#### 2.3.2. In Vivo

A number of researchers have used middle cerebral artery occlusion (permanent or transient) to study focal cerebral ischemia in mice or rats. A study indicated that that baicalin treatment reduced MCAO-induced neuronal damage, brain edema, and blood–brain barrier (BBB) permeability by inhibiting the expression of matrix metallopeptidase 9 (MMP-9) and MMP-9-mediated occludin degradation [63]. Baicalin also showed a protective effect against MCAO-induced cerebral ischemia in rat by reducing the expression of TLR2/4, NF-κB, inducible nitric oxide synthase (iNOS), and COX-2. In addition, baicalin administration decreased the serum content of TNF-α and IL-1β [64]. In another study, baicalin markedly reduced the enzymatic activity of myeloperoxidase (MPO) and downregulated the expression of iNOS and COX-2 mRNAs, and cleaved caspase-3 protein in MCAO-induced rats [59]. In addition, baicalin downregulated the expression of NOD2 and TNFα proteins in bilateral common carotid artery ligation (BCCL)-induced mice [34]. Xue et al. [61] found that baicalin treatment decreased the level of NF-κB p65 in MCAO-induced focal cerebral ischemia in rats. In gerbils with transient global cerebral ischemic/reperfusion injury, the administration of baicalin significantly attenuated ischemia-induced neuronal cell damage in gerbils. In addition, baicalin reduced the level of MDA, and increased SOD, GSH, and GSH-Px activities. Moreover, baicalin markedly upregulated the expression of BDNF and suppressed the expression of caspase-3 at mRNA and protein levels [62].

Zhang et al. [55] compared the individual and combined effects of baicalin with jasminoidin on cerebral ischemia/reperfusion injury in rats. When compared with individual treatment, the combined treatment significantly ameliorated the results of 2,3,5-triphenyltetrazolium chloride (TTC) and histological examination. In addition, this combination ameliorated diffusion weighted imaging (DWI) of magnetic resonance imaging (MRI) and promoted brain-derived neurotrophic factor (BDNF) expression and inhibited caspase-3 expression. The capillary electrophoresis-laser-induced fluorescence detection technique was used to investigate the effect of baicalin on changes of amino acid neurotransmitters level during cerebral ischemia in rats. In the results, concentrations of Glu, Asp, gamma-aminobutyric acid (GABA), and Gly in the brain cortex were elevated due to cerebral ischemia. However, baicalin treatment could attenuate the elevations of Glu and Asp in the cerebral cortex [51]. A study also attempted to investigate and determine the effect of baicalin on different protein expression modes in MCAO-induced mice brains. However, there was no significant difference in the expression of twenty-four proteins between baicalin-treated MCAO group and the sham-operation group [60].

In ischemia/reperfusion to the rat retina, baicalin treatment regulated the localization of Thy-1 and choline acetyltransferase (ChAT) level, and the level of various proteins and mRNAs in the retina. However, baicalin treatment did not affect the level of caspase-8 and caspase-3 mRNAs caused by ischemia/reperfusion [32]. Chang et al. [56] examined the prophylactic effect of baicalin in an animal model of heatstroke. Baicalin administration effectively attenuated the hyperthermia, intracranial hypertension, and increased NO_2_^−^, glutamate, glycerol, lactate/pyruvate ratio, and dihydroxybenzoic acid levels in the hypothalamus of heatstroke-induced rats. Further, baicalin markedly decreased the levels of IL-1β and TNF-α in the serum, as well as the hypothalamus.

In intracerebral hemorrhage (ICH)-induced rats, baicalin significantly attenuated brain edema, inhibited cell apoptosis, and suppressed the expression of brain protease-activated receptor-1 (PAR-1) at both the mRNA and protein levels [70]. Baicalin could also improve neurological function and decrease brain infarction in MCAO-induced rats. Further, baicalin reduced cell apoptosis and the production of ROS and MDA. Moreover, baicalin interfered with SOD and nicotinamide adenine dinucleotide 2′-phosphate oxidase (NOX) activities [38]. Dai et al. [71] investigated the effect of baicalin on global ischemia-induced gerbils, and found that baicalin treatment effectively facilitated neurological function and attenuated the ischemia-induced neuronal damage. Additionally, baicalin administration increased the expression of GABA_A_ receptor (GABA(A)R) α1, GABA(A)R γ2, and K–Cl co-transporter 2 (KCC2), and decreased the expression of Na–K–Cl cotransporter 1 (NKCC1) in the hippocampus of gerbils following an ischemic insult. Moreover, baicalin treatment markedly increased the protein expressions of HSP70 and phosphorylated ERK (p-ERK), and decreased the expression of phosphorylated JNK (p-JNK) and phosphorylated p38 (p-p38) in ischemic gerbils. Baicalin also inhibited 3-nitrotyrosine formation, decreased infarct size, and attenuated apoptotic cell death in ischemia/reperfusion-induced injury in rat brain [40].

A study reported that baicalin treatment increased the number of newly generated cells in the hippocampus of rats exposed to transient cerebral ischemia. Additionally, baicalin improved cognitive impairment as measured in the Morris water maze test [42]. In a rat model of collagenase VII-induced ICH, baicalin administration reduced brain edema, inhibited NF-κB activation, and suppressed MMP-9 expression. In addition, baicalin reduced the production of IL-1β and IL-6, as well as BBB permeability [77]. Baicalin effectively inhibited neuronal apoptosis by enhancing the transcription and expression of MCL-1 and BCL-2 in MCAO-induced rats. In addition, baicalin administration increased MRTF-A level in ischemic hemisphere [21]. Baicalin also improved learning and memory dysfunction in global cerebral ischemia/reperfusion-induced gerbils by downregulating the phosphorylation level of Ca^2+^/calmodulin-dependent protein kinase II (CaMKII) and preventing hippocampal neuronal apoptosis [46]. In a recent study, baicalin administration significantly decreased the mortality rates, and ameliorated BBB disruption and hemorrhagic transformation. In addition, baicalin markedly scavenged peroxynitrite and inhibited MMP-9 expression and activity in MCAO-induced ischemic brains with delayed tissue plasminogen activator treatment [86]. The effect of baicalin on hyperglycemia-exacerbated cerebral ischemia/reperfusion injury in rats was investigated by Li et al. [13]. In their study, baicalin treatment decreased blood glucose, alleviated neurological deficit and reduced infarct volume.

Inflammatory responses and blood–brain barrier disruption play a critical role in the formation of edema during subarachnoid hemorrhagic brain injury. The protective effect of baicalin against filament perforation-induced subarachnoid hemorrhagic brain injury in mice was investigated by Shi et al. [88]. Baicalin administration effectively increased neurological score and brain water content. Baicalin also restored levels of tight junction proteins, such as survivin, claudin-5, zonula occludens-1 (ZO-1), and collagen IV. In addition, baicalin treatment inhibited the production of IL-1β, IL-6, and chemokine (C–X–C motif) ligand 3 (CXCL-3) in subarachnoid hemorrhage mice. Furthermore, baicalin downregulated the mRNA and protein levels of NOS-2 and NOX-2 in subarachnoid hemorrhage mice. Recently, the neuroprotective effect of baicalin in a rat model of hypoxic-ischemic encephalopathy was investigated. In the results, baicalin markedly reduced apoptosis and increased the expression of phosphorylated protein kinase B (p-Akt) and glutamate transporter 1. Further, the PI3K/Akt inhibitor, LY294002 blocked the effect of baicalin on p-Akt and glutamate transporter 1 [89].

### 2.4. Neuroprotective and Cognitive Enhancement Effects

#### 2.4.1. In Vitro

Ketamine is extensively used as an anesthetic in pediatric clinical practice. However, a number of studies have demonstrated that exposure to ketamine during the developmental period induces neuronal toxicity. In vitro experiments showed that baicalin attenuated ketamine-induced cell viability decrease, morphological change, and caspase-3 expression activation in the primary neuron–glia mixed cultures. Further, the results demonstrated that baicalin showed the neuroprotective effect against ketamine-induced toxicity by activating PI3K/Akt and its downstream cAMP response element-binding protein (CREB)/BDNF/Bcl-2 signaling pathways [16]. Baicalin also suppressed co-listin sulfate-induced neuronal apoptosis in PC12 cells by inhibiting free radical injury, and downregulating caspase-3 and lactate dehydrogenase activities [39]. Additionally, baicalin showed a potent neuroprotective effect against H_2_O_2_-induced toxicity in PC12 cells. Baicalin pretreatment of PC12 cells reduced the viability loss and apoptotic rate in H_2_O_2_-induced PC12 cells. In addition, baicalin increased SOD, GSH-Px activities and decreased the level of MDA. Moreover, baicalin upregulated the expression of survivin, Bcl-2, and p-STAT3, and downregulated the expression of caspase-3 expression [15]. In thrombin-induced injury in SH-SY5Y cells, baicalin reduced cell death by inhibiting NF-κB activation and suppressing the expression of PAR-1 and caspase-3 [12].

Prolyl oligopeptidase (POP) is a cytosolic serine peptidase that has been related to schizophrenia, bipolar affective disorder, and associated neuropsychiatric disorders. An in vitro study demonstrated that baicalin inhibited the generation of prolyl oligopeptidase in a dose-dependent manner [33]. STAT3 and basic helix–loop–helix (bHLH) gene family are main cellular signaling molecules to regulate cell fate decision and neuronal differentiation of neural stem/progenitor cells (NSPCs). In this context, Li et al. [37] examined the effects of baicalin on stat3 phosphorylation, the expression of bHLH family proteins, and neuronal differentiation of NSPCs. In their study, baicalin treatment promoted the number of microtubule-associated protein (MAP-2) positive-staining cells, and reduced glial fibrillary acidic protein (GFAP)-staining cells. Additionally, baicalin suppressed the expression of p-stat3 and Hes1, and upregulated the expression of NeuroD1 and Mash1.

In another study, Morita et al. [44] determined whether baicalin promotes neuronal differentiation in human iPS cells, and the expression of bHLH gene during neuronal differentiation. The results demonstrated that baicalin can influence the neuronal fate decision in human iPS cells by promoting neuronal differentiation and inhibiting glial differentiation. In addition, baicalin treatment decreased the level of Hes1 protein and upregulated the expression of Ascl1 gene. The proliferation and migration of Schwann cells (SCs) are important events in the process of peripheral nerve repair. A recent study indicated that baicalin could promote the viability of RSC96 SCs and exhibit the greatest gene expression of neurotrophic factors, such as glial cell-derived neurotrophic factor (GDNF), BDNF, and ciliary neurotrophic factor (CNTF). These neurotrophic factors are considered essential factors in the process of never cell regeneration [49].

#### 2.4.2. In Vivo

In rats with spinal cord injury (SCI), BC treatment remarkably decreased the water content of spinal cord tissue, the permeability of blood–spinal cord barrier, and oxidant stress. Further, baicalin decreased the expression of TNF-α, NF-κB, Bax, Bcl-2, and caspase-3 [20]. Baicalin exhibited a strong neuroprotective activity against ketamine-induced apoptotic neurotoxicity in rat pups. Baicalin treatment increased the expression of p-Akt, phosphorylated-glycogen synthase kinase-3 beta (p-GSK-3β), p-CREB, and BDNF in ketamine-induced rats. In addition, baicalin increased the expression of bcl-2/Bax and decreased the expression of caspase-3 in ketamine-induced rats [16]. The administration of baicalin also enhanced memory impairment as measured in the passive avoidance Morris water maze tests in corticosterone-induced rats. Additionally, the administration of baicalin effectively alleviated memory-related decreases in the expression levels of BDNF and CREB in the hippocampus [75]. Ma et al. [81] found that baicalin exhibited a strong protective activity against streptozotocin-induced diabetes-associated cognitive deficits in rats. The results revealed that baicalin treatment improved memory performances and increased the neuronal survival in streptozotocin-induced rats. Baicalin treatment also increased ChAT, p-ERK, BDNF, and Bcl-2. Furthermore, baicalin decreased the levels of hippocampal acetylcholinesterase (AchE), p-JNK, p-p38, caspase-3, Bax, and plasma glucose.

### 2.5. Epilepsy: In Vivo

Oxidative stress can markedly alter neuronal function, and has been associated with status epilepticus. Liu et al. [67] investigated whether baicalin could exhibit anticonvulsant and neuroprotective effects in pilocarpine-induced epileptic rats. Baicalin treatment remarkably postponed the onset of the first limbic seizures and status epilepticus, reduced the mortality rate, and decreased the changes in the levels of lipid peroxidation, and nitrite and reduced glutathione contents in the hippocampus. Moreover, baicalin attenuated the neuronal cell damage, apoptosis, and degeneration in pilocarpine-induced seizures in the hippocampus of rats. In kainic acid-induced epileptic mice, baicalin administration effectively attenuated neuronal injury and apoptosis in the hippocampus. Further, baicalin decreased the expression of miR-497 and cleaved caspase-3 protein, and upregulated the expression of Bcl-2 protein [83].

### 2.6. Antidepressant Effect: In Vivo

In depressive disorders, the regulation of α-amino-3-hydroxy-5-methyl-4-isoxazolepropionic acid (AMPA) receptor expression plays a key role in the viability of neurons and the level of BDNF in the brain. In chronic unpredictable mild stress (CMS)-induced rats, baicalin administration significantly increased AMPA receptor expression and decreased neuron apoptosis [76]. A study was also carried out to investigate the effect of baicalin on CMS-treated rats. The results demonstrated that baicalin effectively reversed the changes in depressive-like behaviors, such as decreased sucrose intake and locomotor activity, and increased immobility time. In addition, baicalin treatment significantly decreased the activation of NLRP3 inflammasome and the levels of IL-1β and IL-6 in rat prefrontal cortex [10]. Baicalin treatment also increased the sucrose preference, decreased serum corticosterone levels, downregulated the activity and expression of COX-2 mRNA, and decreased the level of prostaglandin E2 (PGE2) in CMS-induced rats [73].

In corticosterone-induced mice, baicalin treatment relieved depressive-like behaviors, including the increased sucrose preference and decreased the duration of immobility. In addition, baicalin restored the level of serum corticosterone, upregulated the expression of glucocorticoid receptor and BDNF, as well as downregulated serum- and glucocorticoid-regulated kinase 1 (SGK1) in the hippocampus. Furthermore, baicalin markedly increased the expression of 11β-hydroxysteroid dehydrogenase-2 (11β-HSD2) protein in the hippocampus [80]. A recent study also demonstrated that baicalin inhibited adaptor protein, phosphotyrosine, interacting with PH domain and leucine zipper 2 (APPL2)-mediated glucocorticoid receptor (GR) hyperactivity, promoted adult neurogenesis, released depressive and anxiety symptoms, and enhanced olfactory functions in chronic corticosterone-induced mice [90]. In a rat model of olfactory bulbectomy-induced depression, Yu et al. [84] found that baicalin effectively increased the performance in depression-like behavioral tests, and decreased oxidative stress, synaptophysin expression, and hippocampal apoptosis. Further, baicalin regulated the levels or activity of MDA, SOD, and GSH-Px. Moreover, baicalin treatment prevented apoptotic PAR-1 expression and suppressed caspase-mediated apoptosis signaling cascades in olfactory bulbectomy-induced rats. In another study, baicalin improved antidepressant-like behaviors in mice and rats. Additionally, oral administration of baicalin effectively inhibited monoamine oxidase A and B activity in rats. Furthermore, baicalin showed a significant recovery in sucrose intake in CMS-induced rats [92]. Zhang et al. [93] found that baicalin treatment improved anxiety/depression-like behaviors and promotes hippocampal neurogenesis in chronic corticosterone-induced mice. Further, baicalin may normalize GR function through serum- and glucocorticoid-inducible kinase 1- and FK506-binding protein 51-mediated GR phosphorylation.

### 2.7. Anxiolytic-Like Effect: In Vivo

Liao et al. [51] found that the anxiolytic-like effect of baicalin may be mediated through the activation of benzodiazepine binding site of GABA(A) receptors. The anxiolytic effects of baicalin alone, and in combination with other anxiolytics in mice, were investigated by Xu et al. [54]. Baicalin treatment showed significant anxiolytic-like effects as measured in the elevated plus-maze test. Co-administration of baicalin with DL-tetrahydropalmatine also exhibited a considerable anxiolytic-like effect. In mice with picrotoxin-induced seizure, Wang et al. [58] suggested that baicalin administration showed anxiolytic-like effects through the alpha2- and alpha3-containing GABA(A) subtypes.

### 2.8. Blood–Brain Barrier Penetrating Ability

The BBB is a highly selective semipermeable membrane barrier, plays an important role in maintaining homeostasis of the CNS. It protects the brain by blocking the passage of medications into the brain. Hence, BBB-penetrating ability is a prerequisite for medications used for the treatment of various brain disorders [45]. Zhang et al. [94] investigated the effect of baicalin on the transport of nimodipine (a dihydropyridine calcium channel blocker) across the BBB. In their study, baicalin (at 2–5 μg/mL) increased the uptake of nimodipine, whereas baicalin (at 10–20 μg /mL) decreased the uptake of nimodipine in rat brain microvascular endothelial cell culture. Liu et al. [95,96] developed a novel brain-targeting drug delivery system based on baicalin-loaded PEGylated cationic solid lipid nanoparticles modified by OX26 antibody (OX26-PEG-CSLN) and demonstrated that OX26-PEG-CSLN improved the uptake of baicalin across the BBB, and enhanced the bioavailability of baicalin in cerebral spinal fluid of rats with cerebral ischemia/reperfusion injury.

Another study demonstrated that intranasal administration of baicalin, about 52.36–100% baicalin content at 8 h, was transported to the brain through the olfactory pathway [97]. Zhang et al. [98] also investigated the pharmacokinetic process of baicalin in normal rat blood, as well as cerebral nuclei, such as cortex, hippocampus, striatum, thalamus, and brain stem. Yang et al. [99] examined the role of Panax notoginsenosides in the pharmacokinetic behavior of baicalin in rat brain. The data indicated that Panax notoginsenosides decreased the elimination rate of baicalin from rat plasma and promoted the penetration of baicalin into rat brain. Additionally, Panax notoginsenosides increased the concentration and reduced the elimination of baicalin from rat brain. A recent study also demonstrated that six *Radix scutellariae* flavones such as baicalein, wogonin, oroxylin A, baicalin, wogonoside, and oroxyloside could cross the blood–brain barrier, with brain concentrations ranging from 7.9 to 224.0 pmol/g [100].

## 3. Conclusions and Future Perspectives

*S. baicalensis* has been traditionally used for the treatment of a variety of disorders since ancient times. Baicalin is the main bioactive component from the root of *S. baicalensis.* The present review summarizes the protective effects of baicalin for the treatment of neurotoxicity-related diseases such as ischemic stroke, AD, and PD. In addition, baicalin exhibits considerable anxiolytic-like and antidepressant effects. In the mechanistic aspects, baicalin shows a strong protective activity against neurotoxicity-mediated disorders by regulating different cell signaling pathways. The published reports reveal that baicalin has antioxidant, anti-inflammatory, anti-apoptotic, and anti-excitotoxicity properties. Interestingly, baicalin promotes neurogenesis and cell differentiation. Baicalin may be a potential candidate for the prevention and treatment of various neurodegenerative diseases and enhancement of cognitive functions due to its multitargeted actions and BBB-penetrating ability. Although baicalin showed potent neuroprotective effects under in vitro and in vivo models, clinical studies in association with baicalin against these diseases are meager. Further studies in relation to safety profiles of baicalin, improvement of its bioavailability and clinical trials are needed.

## Figures and Tables

**Table 1:** Neuroprotective effects of baicalin under in vitro and ex vivo models.

S. No.	Model	Mechanism	Dose	Reference
1	Oxygen/glucose deprivation (OGD)- and *N*-methyl-d-aspartate (NMDA)-induced injuries in rat hippocampal slices	Inhibited viability reduction and acute neuron swelling. Inhibited the increased membrane portion of PKC(α).	0.1, 1, and 10 μM/L	[30]
2	OGD-induced ischemic-like injury in rat cortical neurons	Attenuated Injuries. Inhibited 5-LOX translocation to the nuclear envelope translocation. Inhibited the production of cysteinyl leukotrienes. Decreased intracellular calcium level.	0.2, 1, and 5 µM	[31]
3	Light-, H_2_O_2_-, and serum deprivation-induced toxicity in RGC-5 cells	Attenuated the negative insult of light, H_2_O_2_, and serum withdrawal to RGC-5 cells.	0.1, 0.5, 1, 5, and 10 μM	[32]
4	Prolyl oligopeptidase (POP) inhibition assay	Inhibited POP in a dose-dependent manner.	20, 50, 100, and 150 µM	[33]
5	OGD-induced toxicity in BC2, PC12 and primary neuron cells	Downregulated the expression of NOD2 and TNFα.	10 μg/mL	[34]
6	Aβ-induced toxicity in SH-SY5Y cells	Inhibited Aβ_1-42_ aggregation. Protected oxidative injuries by decreasing H_2_O_2_ production.	2.5, 5, and 10 μM	[35]
7	OGD-induced toxicity in rat microglial cells	Suppressed the release of TNF-α, IL-1β, IL-6, and IL-8. Downregulated TLR4 mRNA expression. Reduced TRAF6 levels. Downregulated the phosphorylation of IκB-α, c-jun, ERK1/2, JNK, p38 and inhibited the transfer of MyD88 from cytoplasm to membrane.	10, 20, and 40 μg/mL	[36]
8	Neural stem/progenitor cells (NSPCs) from the cortex of embryonic E15–16 rats	Increased the percentages of MAP-2 positive-staining cells and decreased GFAP staining cells. Downregulated the expression of p-stat3 and Hes1. Upregulated the expressions of NeuroD1 and Mash1.	2 and 20 μM	[37]
9	H_2_O_2_-induced toxicity in primary rat cortical neurons	Attenuated neuronal injury and improved superoxide dismutase (SOD) activity.	10, 20, 40, 80, and 200 µM	[38]
10	Colistin sulfate-induced toxicity in PC12 cells	Changed the cell morphology and increased the cell viability. Decreased caspase-3 activity, tate dehydrogenase level, and free radical content.	25, 50, and 100 μg/mL	[39]
11	Peroxynitrite-mediated toxicity in SH-SY5Y cells	Protected the neuronal cell damage.	5, 20, and 50 μM	[40]
12	OGD-induced injury in brain microvascular endothelial cells	Increased cell viability, decreased the rate of LDH leakage, and the levels of TNF-α, IL-1β, and IL-6. Downregulated the phosphorylation of MAPK signaling pathway, such as p-MRK1/2, p-ERK and p-p38. Suppressed the phosphorylation of NF-кB signaling pathway, such as p-IKKα, p-IKKβ, and p-IκBα. Inhibited nuclear transcriptional activity triggered via NF-κB p65 and p-IκBα.	10 and 100 μM	[41]
13	NSPCs from rats	Transient exposure of NSPCs to baicalin during proliferation activated Mash1.	7.5, 15, and 30 µM	[42]
14	C6 glioma cells	Downregulated iron concentration by positively regulating divalent metal transporter 1 expression and negatively regulating ferroportin 1 expression. Decreased iron accumulation in the substantia nigra.	100 μg/mL	[43]
15	Aβ_42_-induced toxicity in BV2 microglial cells	Reduced the expression of CD11b, decreased chemotactic ability, and inhibited the secretion of IL-6, TNF-α, and NO. Suppressed the phosphorylation of JAK2 and STAT3.	50 and 100 μM	[14]
16	H_2_O_2_-induced toxicity in PC12 cells	Reduced the viability loss and apoptotic rate. Increased SOD, GSH-Px activities, and decreased MDA level. Increased the expression of survivin, Bcl-2, and p-STAT3, and decreased caspase-3 expression.	1, 2, and 5 µM	[15]
17	Thrombin-induced toxicity in SH-SY5Y cells	Reduced cell death by inhibiting NF-κB activation and suppressing PAR-1 expression. Reduced caspase-3 expression.	5, 10, and 20 μM	[12]
18	Neuronal differentiation of human iPS cells	Promoted neuronal differentiation and inhibited glial differentiation. Reduced Hes1 protein levels and upregulated Ascl1 gene expression.	10 mmol/L	[44]
19	H_2_O_2_-induced toxicity in primary rat cortical neurons	Inhibited neuronal apoptosis by enhancing the transcription and expression of MCL-1 and BCL-2. Increased MRTF-A level. The anti-apoptosis effect of baicalin was inhibited by small interfering RNA of MRTF-A. Enhanced the transactivity of MCL-1 and BCL-2 promoter. LY294002 (PI3K inhibitor) and PD98059 (ERK1/2 inhibitor) reduced baicalin-induced MRTF-A expression and transactivity and expression of MCL-1 and BCL-2.	0.7, 1.4, and 2.8 µM	[21]
20	OGD/reoxygenation (RO)-induced toxicity in SH-SY5Y cells	Attenuated apoptotic cell death. Decreased caspase-3 expression. Downregulated of NF-κB and NMDAR1.	1, 5, and 25 μmol/L	[45]
21	OGD-induced toxicity in hippocampal neurons and SH-SY5Y cells	Suppressed the phosphorylation level of CaMKII.	1 µM	[46]
22	Ketamine-induced toxicity in primary rat cortical neuron–glia mixed cultures	Alleviated cell viability decrease, morphological change, and caspase-3 expression increase.	20, 50, and 100 µM	[16]
23	Cobalt chloride-induced toxicity in PC12 cells	Baicalin–berberine complex showed protective effects	15 µg/mL	[47]
24	OGD/reperfusion-induced toxicity in PC12 cells	Inhibited Drp-1 expression, decreased mitochondrial fission, promoted MFN2 generation, increased Drp-1 Ser637 phosphorylation, and elevated mitochondrial membrane potential. Suppressed cell apoptosis and enhanced mitophagy.	0.1, 1, 10, and 20 µM	[13]
25	OGD-induced toxicity in microvascular endothelial cells from mouse brain	Inhibited cell death, reduced cell membrane damage, and maintained the integrity of the nucleus. Decreased the necroptosis ratio. Regulated the expression of RIP-1 and RIP-3 in bEnd.3 cells. Inhibited the production of ROS and malondialdehyde. Increased the activity of SOD.	100 and 200 μM	[48]
26	Schwann cells (SCs) of RSC96	Promoted the viability of RSC96 SCs and gene expression of GDNF, BDNF, and CNTF.	5, 10, and 20 μM	[49]
27	Dopamine-induced minimal hepatic encephalopathy in primary hippocampal neurons	Blocked dopamine-induced reduction of GABA_A_R levels. Improved the interaction of GABA_A_R with TrkB. Prevents dopamine-induced impairment of synaptogenesis.	1, 2.5, 5, 10, and 30 μM	[50]

**Table 2:** Neuroprotective effects of baicalin under in vivo models.

S. No.	Model	Mechanism	Dose	References
1	Middle cerebral artery occlusion (MCAO)-induced focal cerebral ischemia in rats	Attenuated the elevations of Glu and Asp.	300 mg/kg, intra-sublingually	[51]
2	Anxiolytic-like effects in mice	Increased the number of shocks as measured in Vogel lick-shock conflict paradigm. Anxiolytic-like effect baicalin was antagonized by a benzodiazepine receptor antagonist, flumazenil (2 mg/kg, ip).	20 mg/kg, ip	[52]
3	MCAO-induced cerebral ischemia in rats	Reduced the infarction areas. Increased the gene expression of RpL19 and Csnk2.	40 mg/kg, po	[53]
4	Anxiolytic-like effect in mice	Increased entries into and time spent in open arms. Improved the performance in the hole-board and horizontal wire tests.	3.75, 7.5, 15, and 30 mg/kg, po	[54]
5	Focal cerebral ischemia–reperfusion injury in rats	Ameliorated the results of TTC and histological examination. Baicalin/jasminoidin combination ameliorated DWI of MRI and behavior examination results. Promoted the expression of BDNF and inhibited the expression of caspase-3.	Baicalin—15 mg/kg, or a combination of baicalin (15 mg/kg) and jasminoidin (15 mg/kg), iv	[55]
6	Heat stress on cerebrovascular and metabolic functions in rats	Improved survival during heatstroke. Reduced the hyperthermia, intracranial hypertension, and increased levels of NO metabolite, glutamate, glycerol, lactate/pyruvate ratio, and dihydroxybenzoic acid in the hypothalamus. Suppressed the levels of IL-1β and TNF-α in the serum and hypothalamus.	10, 20, and 40 mg/kg, iv	[56]
7	MPTP-induced toxicity in mice	Decreased score in the hanging and swimming tests. Prevented the loss of TH-positive neurons and the decrease of dopamine content. Increased the content of GSH in the brain.	100 mg/kg, po	[57]
8	Picrotoxin-induced seizure in mice	Improved behavioral performances as measured in step-through passive avoidance and rotarod tests. Showed preference for alpha2- and alpha3-containing GABA(A) subtypes.	3.3, 10, and 30 mg/kg, po	[58]
9	Ischemic insult to retina of one eye of a rat	Regulated the localization of Thy-1 and ChAT, and the content of various proteins and mRNAs.	12.5 mg/kg, ip	[32]
10	MCAO-induced focal cerebral ischemia in rats	Reduced neurological deficit scores and cerebral infarct volume. Decreased the enzymatic activity of MPO and the expression of iNOS and COX-2. Inhibited neuronal apoptosis and the expression of cleaved caspase-3 protein.	10, 30, and 100 mg/kg, ip	[59]
11	MCAO-induced focal cerebral ischemia in mice	Performed well in regulating proteins in energy metabolism.	20 mg/kg, i.v	[60]
12	Spinal cord injury (SCI) in rat	Decreased the water content of spinal cord tissue, the permeability of blood–spinal cord barrier, oxidant stress. Downregulated the expression of TNF-α, NF-κB, Bax, Bcl-2, and caspase-3. Improved the recovery of limb function.	10, 30, and 100 mg/kg, ip	[20]
13	Bilateral common carotid artery ligation (BCCL)-induced cerebral ischemia in rats	Downregulated the expression of NOD2 and TNFα in protein levels.	10, and 50 mg/kg, ip	[34]
14	MCAO-induced focal cerebral ischemia in rats	Decreased neurological deficit scores and reduced the volume of infarction. Decreased the level of NF-κB p65.	50, 100, and 200 mg/kg, ip	[61]
15	Occlusion of common carotid arteries-induced ischemia in gerbils	Attenuated neuronal cell damage. Reduced the level of MDA. Elevated SOD, GSH, and GSH-PX activities. Promoted the expression of BDNF and inhibited the expression of caspase-3.	50, 100, and 200 mg/kg, ip	[62]
16	MCAO-induced focal cerebral ischemia in rats	Reduced the neuronal damage, brain edema, and blood–brain barrier (BBB) permeability. Downregulated the expression of MMP-9 protein and mRNA. Upregulated the expression of occludin.	100 mg/kg, ip	[63]
17	MCAO-induced cerebral ischemia in rats	Reduced cerebral infarct area and infarct volume. Decreased the expression of TLR2/4, NF-κB, iNOS, and COX-2. Attenuated TNF-α and IL-1β levels.	100 mg/kg, ip	[64]
18	Global ischemia/reperfusion injury in rats	Improved learning and memory. Decreased hippocampal apoptosis and reduced the level of COX-2 expression.	100 mg/kg, po	[65]
19	4-vessel occlusion-induced global ischemic model in rat	Inhibited the hippocampal neuronal cell death.	10 mg/kg, po	[66]
20	Pilocarpine-induced epileptic model in rats	Delayed the onset of the first limbic seizures and status epilepticus. Reduced the mortality rate, and attenuated the changes of lipid peroxidation, nitrite content, and reduced glutathione levels. Attenuated the neuronal cell loss, apoptosis, and degeneration.	100 mg/kg, ip	[67]
21	BCCL-induced cerebral ischemia-reperfusion in rats	Prolonged the terminal half-life of baicalin	90 mg/kg, iv	[68]
22	Rotenone-induced Parkinson’s disease in rats	Inhibited iron accumulation in different brain regions.	78 mg/kg, po	[69]
23	Collagenase VII-induced ICH in rats	Attenuated brain edema and inhibited cell apoptosis. Suppressed the expression of PAR-1.	25, 50, or 100 mg/kg, ip	[70]
24	MCAO-induced cerebral ischemia in rats	Improved neurological function and decreased brain infarction. Reduced cell apoptosis and inhibited the production of ROS and MDA. Interfered with SOD and NOX oxidase activities.	15 mg/kg, iv	[38]
25	Bilateral common carotid arteries (BCCA)-induced global ischemia/reperfusion injury in gerbils	Facilitated neurological function and suppressed neuronal damage. Increased GABA(A)R α1, GABA(A)R γ2 and KCC2. Decreased NKCC1 level. Upregulated the protein expressions of HSP70 and p-ERK, and diminished the expression of p-JNK and p-p38.	200 mg/kg, ip	[71]
26	MCAO-induced cerebral ischemia in mice	Out of the 10 most significant molecular functions, 7 were common to baicalin and controls, and only 3 occurred in baicalin group.	5 mg/kg, iv	[72]
27	Chronic unpredictable mild stress (CMS)-induced depressive-like behavior in rats	Prevented the abnormalities induced by CMS. Decreased COX-2 activity and expression, and reduced the level of PGE2.	10, 20, and 40 mg/kg, po	[73]
28	MCAO-induced cerebral ischemia injury in rats	Inhibited the formation of 3-nitrotyrosine, reduced infarct size, and attenuated apoptotic cell death.	10, 25, and 50 mg/kg, iv	[40]
29	Ibotenic acid-induced dementia in rats	A combination of three chemicals attenuated abnormalities in cognition, brain functional images, and brain histological morphology. Influenced the expression levels of 19 genes in the forebrain.	3 mL/kg, po (1.25 mg/mL baicalin, 6.25 mg/mL jasminoidin, and 1.75 mg/mL cholic acid)	[74]
30	BCCA-induced transient cerebral ischemia in rats	Increased the number of newly generated cells and promoted new neuron production. Improved cognitive impairment in Morris water maze test.	50 mg/kg, ip	[42]
31	Chronic corticosterone-induced learning and memory deficits in rats	Improved memory impairment in the passive avoidance test and reduced the escape latency in the Morris water maze test. Upregulated the expression of BDNF and CREB.	20, 50, and 100 mg/kg, ip	[75]
32	AD transgenic mice (APPswe, PSEN1de9)	Inhibited microglial cell activation by regulating the JAK2/STAT3 signaling pathway.	100 mg/kg, po	[14]
33	Rats induced with CMS	Increased AMPA receptor expression and decreased neuron apoptosis.	20, and 40 mg/kg, po	[76]
34	Collagenase VII-induced ICH in rats	Reduced brain edema, inhibited NF-κB activation, and suppressed MMP-9 expression. Reduced IL-1β and IL-6 production, and BBB permeability.	25, 50, and 100 mg/kg, ip	[77]
35	Aβ-induced AD in mice	Ameliorated memory impairment in the Morris water maze and probe tests. Attenuated glial cell activations, and increase of TNF-α and IL-6 expressions.	30, 50, and 100 mg/kg, po	[78]
36	Aβ_1–42_-induced learning and memory deficits in rats	Improved learning and memory deficits. Attenuated the hippocampus injury caused by Aβ. Increased SOD, catalase, and GSH-px activities and upregulated their gene expression. Increased mitochondrial membrane potential, and decreased Bax/Bcl-2 ratio, cytochrome c release, and caspase-9/-3 activation. Activated Nrf2 signaling.	50, 100, and 200 mg/kg, i.p	[79]
37	Corticosterone-induced depressive-like behaviors in mice	Increased sucrose preference and decreased duration of immobility. Downregulated the mRNA and protein expression of glucocorticoid receptor and BDNF. Upregulated the serum- and SGK1 in the hippocampus. Increased the expression of 11β-HSD2 protein in the hippocampus.	10, and 20 mg/kg, po	[80]
38	Streptozotocin-induced diabetes-associated cognitive deficits in rats	Improved memory performances and neuronal survival. Increased ChAT, p-ERK, BDNF, and Bcl-2. Downregulated the levels of hippocampal AChE, p-JNK, p-p38, caspase-3, Bax, and plasma glucose.	50, 100, and 200 mg/kg, ip	[81]
39	MCAO-induced ischemia/reperfusion in rats	Inhibited neuronal apoptosis and enhanced transcription and expression of MCL-1 and BCL-2. Increased myocardin-related transcription factor-A (MRTF-A) level in ischemic hemisphere.	50, 100, and 200 mg/kg, po	[21]
40	MCAO-induced reperfusion in mice	Targeted pathways associated with development, neurophysiological processes, and cytoskeleton remodeling.	20 mg/kg, iv	[82]
41	Kainic acid-induced epileptic mice	Attenuated neuronal damage and apoptosis in the hippocampus. Decreased the expression of miR-497 and cleaved caspase-3 protein. Upregulated the expression of Bcl-2 protein.	100 mg/kg, ip	[83]
42	Global cerebral ischemia in gerbils	Improved learning and memory dysfunction by downregulating the phosphorylation level of CaMKII.	100 mg/kg, ip	[46]
43	Ketamine-induced toxicity in rats	Alleviated morphological change and apoptosis. Downregulated caspase-3 activity and caspase-3 mRNA expression. Inhibited p-Akt and p-GSK-3β decrease, and relieved p-CREB and BDNF expression decrease. Increased Bcl-2/Bax and decreased caspase-3 expression.	25, 50, and 100 mg/kg, ip	[16]
44	Olfactory bulbectomy-induced depression in rats	Increased the performance in depression-like behavioral tests. Decreased oxidative stress, synaptophysin expression, and hippocampal apoptosis. Modulated the levels MDA, SOD, and GSH-Px. Prevented apoptotic protease-activating factor-1 expression. Suppressed caspase-mediated apoptosis signaling cascades.	20, and 40 mg/kg, po	[84]
45	In triple-transgenic (3xTg-AD) mice	Flavocoxid (a mixture of purified baicalin and catechin) improved learning and memory function. Decreased eicosanoid production and reduced the phosphorylation level of APP-p-Thr668, p-Thr181 and p-ERK, and the activation of the NLRP3 inflammasome.	20 mg/kg, ip	[85]
46	MCAO-induced cerebral ischemia in rats	Reduced the mortality rates, ameliorated the tissue plasminogen activator-mediated BBB disruption and hemorrhagic transformation. Scavenged peroxynitrite and inhibited MMP-9 expression.	50, 100, and 150 mg/kg, femoral vein	[86]
47	Hyperglycemia-exacerbated MCAO-induced ischemia/reperfusion in rats	Reduced blood glucose, relieved neurological deficit, and decreased infarct volume.	100 mg/kg	[13]
48	CMS-induced rats	Reversed the changes of depressive-like behavior. Decreased the activation of NLRP3 inflammasome and IL-1β and IL-6 levels.	20 and 40 mg/kg	[10]
49	MCAO-induced cerebral ischemia in mice	Reduced the ischemic infarct volume. BA resulted in targeting of pathways related to development, G-protein signaling, apoptosis, signal transduction, and immunity.	5 mg/mL, iv	[87]
50	Subarachnoid hemorrhagic brain injury in mice via filament perforation	Restored the level of tight junction proteins such as occludin, claudin-5, ZO-1, and collagen IV. Inhibited the production of IL-1β, IL-6, and CXCL-3. Attenuated the induction of NOS-2 and NOX-2.	100 mg/kg, ip	[88]
51	Left common carotid artery ligation followed by hypoxia in rats	Reduced cerebral infarct volume and neuronal loss. Inhibited apoptosis, and upregulated the expression of p-Akt and glutamate transporter 1.	120 mg/kg, ip	[89]
52	Dopamine-induced minimal hepatic encephalopathy in rats	Reversed the inactivation of the GABA(A)Rβ/TrkB signaling pathway. Prevented the impairment of synaptogenesis and improved the memory performance.	20, 50, and 100 mg/kg, ip	[50]
53	Chronic corticosterone-induced depression in mice	Inhibited APPL2-mediated GR hyperactivity and promoted adult neurogenesis. Released depressive and anxiety symptoms and enhanced olfactory functions.	3.35 and 6.7 mg/kg, po	[90]
54	Aβ_1-40_-induced Alzheimer’s disease in rats	The percentages of astrocytes and neurons were increased. Enhanced the expressions of Nestin and nucleotide sugar epimerase.	10 mg/kg, ip	[91]
55	CMS-induced depressant-like effect in mice and rats	Reduced immobility time in tail suspension test and the forced swimming test in mice. Decreased immobility time in forced swimming test in rats. Showed a significant recovery in sucrose intake. Inhibited monoamine oxidase A and B activity in a dose-dependent manner in rats.	Mice—10, 20, 40, 60, and 80 mg/kg, po Rats—6.25, 12.5, 25, 50, and 100 mg/k, po	[92]
56	Chronic corticosterone-induced anxiety/depression in mice	Alleviated several anxiety/depression-like behaviors. Increased Ki-67- and DCX-positive cells. Normalized the chronic corticosterone-induced decrease in GR protein levels, the increase in GR nuclear translocation, and the increase in GR phosphorylation at Ser203 and Ser211. Further, regulated the level of FK506-binding protein 51 and phosphorylated serum- and glucocorticoid-inducible kinase 1 at Ser422 and Thr256.	40, 80, and 160 mg/kg, po	[93]

## Abbreviations

11β-HSD2	11β-hydroxysteroid dehydrogenase-2
5-LOX	5-lipoxygenase (5-LOX)
AchE	acetylcholinesterase
AD	Alzheimer’s disease
Akt	protein kinase B
AMPA	α-amino-3-hydroxy-5-methyl-4-isoxazolepropionic acid
APP	amyloid precursor protein
APPL2	adaptor protein, phosphotyrosine interacting with PH domain and leucine zipper 2
Aβ	amyloid-β
BBB	blood–brain barrier
BCCA	bilateral common carotid artery
BCCL	bilateral common carotid artery ligation
Bcl-2	B cell lymphoma 2 family
BDNF	brain-derived neurotrophic factor
bHLH	basic helix–loop–helix
CaMKII	Ca^2+^/calmodulin-dependent protein kinase II
CD11b	cluster of differentiation molecule 11b
ChAT	choline acetyltransferase
CMS	chronic mild stress
CNS	central nervous system
CNTF	ciliary neurotrophic factor
COX-1/2	cyclooxygenases-1/2
CREB	cAMP response element-binding protein
CXCL3	chemokine (C–X–C motif) ligand 3 (CXCL-3)
Drp-1	dynamin-related protein 1
DWI	diffusion weighted imaging
ERK1/2	extracellular signal-regulated kinases 1 and 2
GABA(A)R	GABA_A_ receptor
GABA	gamma-aminobutyric acid
GDNF	glial cell-derived neurotrophic factor
GFAP	glial fibrillary acidic protein
GR	glucocorticoid receptor
GSH-Px	glutathione peroxidase
GSH	glutathione
GSK-3β	glycogen synthase kinase 3 beta
H_2_O_2_	hydrogen peroxide
HSP-70	heat shock protein-70
IL-6	interleukin 6
IL-β	interleukin beta
iNOS	inducible nitric oxide synthase
IκB-α	nuclear factor of kappa light polypeptide gene enhancer in B cells inhibitor-alpha
JAK2	Janus kinase 2
JNK	c-Jun N-terminal kinase
KCC2	K–Cl co-transporter 2
LDH	lactate dehydrogenase
MAP-2	microtubule-associated protein
MAPK	mitogen-actives protein kinases
MCAO	middle cerebral artery occlusion
MCL-1	myeloid cell leukemia-1
MDA	malondialdehyde
MFN2	mitochondrial fission, elevated mitofusin-2
MMP-9	matrix metallopeptidase 9
MPO	myeloperoxidase
MPTP	1-methyl-4-phenyl-1,2,3,6-tetrahydropyridine
MRI	magnetic resonance imaging
MRTF-A	myocardin-related transcription factor-A
MyD88	myeloid differentiation primary response 88
NF-κB	nuclear factor kappa-light-chain-enhancer of activated B cells
NKCC1	Na–K–Cl cotransporter-1
NLRP3	nucleotide-binding domain and leucine-rich repeat protein-3
NMDA	*N*-methyl-d-aspartic acid
NMDAR1	*N*-methyl-d-aspartic acid receptor-1
NO	nitric oxide
NOD2	nucleotide-binding oligomerization domain protein 2
NOX	nicotinamide adenine dinucleotide 2′-phosphate oxidase
Nrf2	nuclear factor erythroid 2-related factor 2
NSPCs	neural stem/progenitor cells
OGD	oxygen/glucose deprivation
OGD/RO	oxygen/glucose deprivation/reoxygenation
PAR-1	protease-activated receptor-1
PD	Parkinson’s disease
PGE2	prostaglandin E2
PI3K	phosphatidylinositide 3-kinase
POP	prolyl oligopeptidase
ROS	reactive oxygen species
SCI	spinal cord injury
SCs	Schwann cells
SGK1	serum- and glucocorticoid-regulated kinase 1
siRNA	small interfering RNA
SOD	superoxide dismutase
STAT3	signal transducer and activator of transcription 3
TH	tyrosine hydroxylase
TLR4	Toll like receptor-4
TNF-α	tumor necrosis factor alpha
TRAF6	tumor necrosis factor receptor-associated factor 6
TRkB	tropomyosin receptor kinase B
TTC	2,3,5-triphenyltetrazolium chloride
ZO-1	zonula occludens-1.
